# Arterolane–piperaquine–mefloquine versus arterolane–piperaquine and artemether–lumefantrine in the treatment of uncomplicated *Plasmodium falciparum* malaria in Kenyan children: a single-centre, open-label, randomised, non-inferiority trial

**DOI:** 10.1016/S1473-3099(20)30929-4

**Published:** 2021-10

**Authors:** Mainga Hamaluba, Rob W van der Pluijm, Joseph Weya, Patricia Njuguna, Mwanajuma Ngama, Peter Kalume, Gabriel Mwambingu, Caroline Ngetsa, Juliana Wambua, Mwanamvua Boga, Neema Mturi, Altaf A Lal, Arshad Khuroo, Walter R J Taylor, Sónia Gonçalves, Olivo Miotto, Mehul Dhorda, Brian Mutinda, Mavuto Mukaka, Naomi Waithira, Richard M Hoglund, Mallika Imwong, Joel Tarning, Nicholas P J Day, Nicholas J White, Philip Bejon, Arjen M Dondorp

**Affiliations:** a KEMRI Wellcome Trust Research Programme, Kilifi, Kenya; b Centre for Tropical Medicine and Global Health, Nuffield Department of Medicine, University of Oxford, Oxford, UK; c MRC Centre for Genomics and Global Health, Big Data Institute, University of Oxford, Oxford, UK; d Mahidol-Oxford Tropical Medicine Research Unit, Faculty of Tropical Medicine, Mahidol University, Bangkok, Thailand; e Department of Molecular Tropical Medicine and Genetics, Faculty of Tropical Medicine, Mahidol University, Bangkok, Thailand; f Sun Pharmaceutical Industries, Gurugram, India; g Asia-Pacific Regional Centre, WorldWide Antimalarial Resistance Network, Bangkok, Thailand; h Wellcome Sanger Institute, Hinxton, UK

## Abstract

**Background:**

Triple antimalarial combination therapies combine potent and rapidly cleared artemisinins or related synthetic ozonides, such as arterolane, with two, more slowly eliminated partner drugs to reduce the risk of resistance. We aimed to assess the safety, tolerability, and efficacy of arterolane–piperaquine–mefloquine versus arterolane–piperaquine and artemether–lumefantrine for the treatment of uncomplicated falciparum malaria in Kenyan children.

**Methods:**

In this single-centre, open-label, randomised, non-inferiority trial done in Kilifi County Hospital, Kilifi, coastal Kenya, children with uncomplicated *Plasmodium falciparum* malaria were recruited. Eligible patients were aged 2–12 years and had an asexual parasitaemia of 5000–250 000 parasites per μL. The exclusion criteria included the presence of an acute illness other than malaria, the inability to tolerate oral medications, treatment with an artemisinin derivative in the previous 7 days, a known hypersensitivity or contraindication to any of the study drugs, and a QT interval corrected for heart rate (QTc interval) longer than 450 ms. Patients were randomly assigned (1:1:1), by use of blocks of six, nine, and 12, and opaque, sealed, and sequentially numbered envelopes, to receive either arterolane–piperaquine, arterolane–piperaquine–mefloquine, or artemether–lumefantrine. Laboratory staff, but not the patients, the patients' parents or caregivers, clinical or medical officers, nurses, or trial statistician, were masked to the intervention groups. For 3 days, oral artemether–lumefantrine was administered twice daily (target dose 5–24 mg/kg of bodyweight of artemether and 29–144 mg/kg of bodyweight of lumefantrine), and oral arterolane–piperaquine (arterolane dose 4 mg/kg of bodyweight; piperaquine dose 20 mg/kg of bodyweight) and oral arterolane–piperaquine–mefloquine (mefloquine dose 8 mg/kg of bodyweight) were administered once daily. All patients received 0·25 mg/kg of bodyweight of oral primaquine at hour 24. All patients were admitted to Kilifi County Hospital for at least 3 consecutive days and followed up at day 7 and, thereafter, weekly for up to 42 days. The primary endpoint was 42-day PCR-corrected efficacy, defined as the absence of treatment failure in the first 42 days post-treatment, of arterolane–piperaquine–mefloquine versus artemether–lumefantrine, and, along with safety, was analysed in the intention-to-treat population, which comprised all patients who received at least one dose of a study drug. The 42-day PCR-corrected efficacy of arterolane–piperaquine–mefloquine versus arterolane–piperaquine was an important secondary endpoint and was also analysed in the intention-to-treat population. The non-inferiority margin for the risk difference between treatments was −7%. The study is registered in ClinicalTrials.gov, NCT03452475, and is completed.

**Findings:**

Between March 7, 2018, and May 2, 2019, 533 children with *P falciparum* were screened, of whom 217 were randomly assigned to receive either arterolane–piperaquine (n=73), arterolane–piperaquine–mefloquine (n=72), or artemether–lumefantrine (n=72) and comprised the intention-to-treat population. The 42-day PCR-corrected efficacy after treatment with arterolane–piperaquine–mefloquine (100%, 95% CI 95–100; 72/72) was non-inferior to that after treatment with artemether–lumefantrine (96%, 95% CI 88–99; 69/72; risk difference 4%, 95% CI 0–9; p=0·25). The 42-day PCR-corrected efficacy of arterolane–piperaquine–mefloquine was non-inferior to that of arterolane–piperaquine (100%, 95% CI 95–100; 73/73; risk difference 0%). Vomiting rates in the first hour post-drug administration were significantly higher in patients treated with arterolane–piperaquine (5%, 95% CI 2–9; ten of 203 drug administrations; p=0·0013) or arterolane–piperaquine–mefloquine (5%, 3–9; 11 of 209 drug administrations; p=0·0006) than in patients treated with artemether–lumefantrine (1%, 0–2; three of 415 drug administrations). Upper respiratory tract complaints (n=26 for artemether–lumefantrine; n=19 for arterolane–piperaquine–mefloquine; n=23 for arterolane–piperaquine), headache (n=13; n=4; n=5), and abdominal pain (n=7; n=5; n=5) were the most frequently reported adverse events. There were no deaths.

**Interpretation:**

This study shows that arterolane–piperaquine–mefloquine is an efficacious and safe treatment for uncomplicated falciparum malaria in children and could potentially be used to prevent or delay the emergence of antimalarial resistance.

**Funding:**

UK Department for International Development, The Wellcome Trust, The Bill & Melinda Gates Foundation, Sun Pharmaceutical Industries

## Introduction

Artemisinin-based combination therapies (ACTs) are first-line drugs for the treatment of uncomplicated falciparum malaria in all malaria endemic countries. ACTs have substantially contributed to the decrease in malaria transmission in the current millennium.^1^ However, artemisinin resistance has emerged and spread in the Greater Mekong subregion in southeast Asia,^2^ followed by rapidly increasing resistance to the ACT partner drugs mefloquine and piperaquine. This resistance has caused high treatment failure in patients with uncomplicated falciparum malaria treated with artesunate–mefloquine (on the Thailand–Burma border)^3^ and dihydroartemisinin–piperaquine (in Cambodia, east Thailand, and Vietnam).^4^ Fit multidrug-resistant malaria parasites could spread to the Indian subcontinent and to sub-Saharan Africa, as has happened in the past with chloroquine and sulfadoxine–pyrimethamine.^5^, ^6^ In addition, artemisinin-resistant *Plasmodium falciparum* can emerge independently in Africa, as it did in Rwanda.^7^ Because novel antimalarial compounds are not likely to be registered within the next 5 years,^8^ strategies that use currently available drugs have to be developed to treat multidrug-resistant falciparum malaria and to delay its spread. These strategies include the use of triple ACTs and the use of non-artemisinin-based triple drug combination therapies. Triple ACTs combine an artemisinin with two partner drugs that are slowly eliminated and have similar pharmacokinetic profiles to each other. Ideally, the two partner drugs in triple ACTs should have different resistance mechanisms, like those between lumefantrine and amodiaquine and between piperaquine and mefloquine.^9^, ^10^, ^11^, ^12^ The triple ACTs artemether–lumefantrine–amodiaquine and dihydroartemisinin–piperaquine–mefloquine have been shown to be efficacious, safe, and well tolerated for the treatment of uncomplicated falciparum malaria, including in regions with high ACT failure rates.^13^ Non-artemisinin-based antimalarials include the synthetic ozonides, arterolane maleate (OZ277) and artefenomel (OZ439). The fixed dose combination arterolane–piperaquine was shown to be an efficacious and safe treatment for *P falciparum* malaria in both children and adults across India and Africa^14^, ^15^ and for *Plasmodium vivax* malaria.^16^ The current dosing scheme for arterolane–piperaquine is age based rather than weight based, resulting in variable dosing per patient weight band. Retrospective analyses of dose finding studies in Africa and Asia indicated that a dose of 4 mg/kg of bodyweight or higher of arterolane is needed for optimal parasite clearance (Tarning J, unpublished). We aimed to assess the safety, tolerability, and efficacy of arterolane–piperaquine–mefloquine versus arterolane–piperaquine and artemether–lumefantrine, the latter being the first-line treatment on the Kenyan coast, in the treatment of uncomplicated falciparum malaria in Kenyan children. In the groups containing arterolane–piperaquine, we used novel dosing schedules aimed at an arterolane dose of 4 mg/kg, while maintaining the same arterolane:piperaquine ratio as in the original formulation.

**Research in context**

**Evidence before this study**

We searched PubMed for articles published in English between database inception and Aug 31, 2020, using the search terms “arterolane” AND “malaria”, which resulted in 34 articles. In addition, we searched PubMed with the same date and language restrictions using the search terms “malaria” AND (“triple ACT” OR “TACT”), which resulted in 35 articles. Arterolane maleate is a synthetic, rapidly acting, potent ozonide antimalarial. The fixed-dose combination of arterolane–piperaquine has been shown to be efficacious and safe for the treatment of *Plasmodium falciparum* and *Plasmodium vivax* malaria in both children and adults across India. There were no studies on triple antimalarial combinations including arterolane–piperaquine. A study in healthy Thai adult volunteers found that exposure to artemisinin dihydroartemisinin was decreased when dihydroartemisinin–piperaquine was combined with mefloquine. The TRACII trial showed that dihydroartemisinin–piperaquine–mefloquine and artemether–lumefantrine–amodiaquine were highly efficacious, safe, and well tolerated in patients with *P falciparum* malaria.

**Added value of this study**

In this study, we show that both arterolane–piperaquine and arterolane–piperaquine–mefloquine are highly efficacious, safe, and well tolerated treatments for uncomplicated falciparum malaria in Kenyan children. The pharmacokinetic profile of arterolane was not affected by the addition of mefloquine to arterolane–piperaquine.

**Implications of all the available evidence**

Deploying arterolane-based triple-combination therapies could delay the development of antimalarial drug resistance against arterolane and its partner drugs, because the chance of parasites developing resistance to all three drugs is the product of the chance of developing resistance to each individual drug.

## Methods

### Study design and participants

We did a single-centre, open-label, randomised, non-inferiority trial with children in Kilifi County Hospital in Kilifi, coastal Kenya (appendix p 1). Participants were recruited from Kilifi County Hospital and the Pingilikani dispensary, a dispensary in Banda ra Salama, Pingilikani Sub-Location, with intermediate-to-high malaria transmission that is about 30 km from Kilifi County Hospital (appendix p 1). Febrile patients presenting at the Pingilikani dispensary were screened by use of a rapid diagnostic test (SD BIOLINE Malaria Ag P.f/Pan; Abbott Diagnostics Korea; Seoul, South Korea) for *P falciparum* malaria. For patients with a positive rapid diagnostic test but no signs of severe or complicated malaria or other disease, written informed consent was obtained from their parent or guardian, after which patients were admitted to Kilifi County Hospital. Patients directly presenting with fever at Kilifi County Hospital were screened for *P falciparum* malaria by use of a rapid diagnostic test and a blood film.

Eligible participants were aged 2–12 years and had uncomplicated *P falciparum* infection, defined as a positive blood smear with asexual forms of *P falciparum* (that might be mixed with non-falciparum species), an asexual parasitaemia of 5000–250 000 parasites per μL, and a fever (a tympanic temperature >37·5°C or a history of fever within the past 48 h before enrolment). They were also able to take oral medication, were willing and able to comply with the study protocol for the duration of the study, and had a parent or guardian provide written informed consent. An independent witness was sought in case of an illiterate parent. The exclusion criteria were: signs of severe or complicated malaria according to WHO guidelines;^17^ the need for immediate treatment with a parenteral antimalarial, as judged by the treating clinician; an acute illness other than malaria requiring urgent systemic treatment, such as antibiotics; a previous splenectomy; treatment with an artemisinin or an ACT in the previous 7 days; treatment with mefloquine in the previous 2 months; a known hypersensitivity or contraindication to any of the study drugs; a QT interval corrected for heart rate (QTc interval) using Bazett's correction method (QTcB interval) of more than 450 ms; a known personal or family history of cardiac conduction problems; or participation in another clinical trial in the previous 3 months.

The protocol was approved by the Oxford Tropical Research Ethics Committee in the UK and the Kenya Medical Research Institute Scientific and Ethics Review Unit in Kenya. The trial was monitored collaboratively by the Mahidol-Oxford Tropical Medicine Research Unit (MORU) and Kenya Medical Research Institute Wellcome Trust Research Programme clinical trials support groups.

### Randomisation and masking

The admitting clinician enrolled all participants, assigned them to the trial groups, and was involved with subsequent patient care and reviews. By use of block sizes of six, nine, and 12, and opaque, sealed, sequentially numbered envelopes, patients were randomly assigned (1:1:1) to receive either arterolane–piperaquine, arterolane–piperaquine–mefloquine, or artemether–lumefantrine. Randomisation sequences were prepared by use of a computer code, with a computer seed included in the program to allow for reproducibility, before the start of the trial by the trial statistician (MM), who conducted the final analysis. Using the randomisation sequences, the envelopes containing treatment allocation information were prepared by the MORU clinical trial support group. All samples were deidentified and the laboratory staff were masked to group assignment. The patients, the patients' parents or caregivers, clinical or medical officers, nurses, and the trial statistician were not masked to the intervention groups. All treatments were directly observed.

### Procedures

For 3 consecutive days, oral artemether–lumefantrine was administered twice daily with a fatty snack or drink (containing at least 2 g of fat) to maximise absorption, whereas oral arterolane–piperaquine and oral arterolane–piperaquine–mefloquine were administered once daily with a non-fatty snack or water.^18^ Artemether–lumefantrine (Coartem; 20 mg of artemether and 120 mg of lumefantrine per tablet; Novartis, Basel, Switzerland) was dosed by bodyweight according to WHO's guidelines for the treatment of malaria (target dose 5–24 mg/kg of bodyweight of artemether and 29–144 mg/kg of bodyweight of lumefantrine) and was administered at hours 0, 8, 24, 36, 48, and 60 (appendix p 12).^19^ The weight-based dosing schedule of arterolane–piperaquine (Synriam; 37·5 mg of arterolane maleate and 187·5 mg of piperaquine phosphate per tablet; Sun Pharmaceuticals, Gurugram, India; administered at hours 0, 24, and 48) aimed for an arterolane dose of 4 mg/kg of bodyweight and a piperaquine dose of 20 mg/kg of bodyweight, matching WHO's recommendations for the dosing of dihydroartemisinin–piperaquine (appendix p 11).^19^ Mefloquine (Lariam; 250 mg per tablet; Roche, Basel, Switzerland) was administered at hours 0, 24, and 48, together with arterolane–piperaquine, aiming for a dose of 8 mg/kg of bodyweight per day. A single low dose of oral primaquine (Centurion Laboratories; Vadodara, India) was administered 24 h after the start of treatment, according to patient age (target dose 0·25 mg/kg of bodyweight; appendix p 12).^20^ Dosing schedules are summarised in the appendix (pp 11–12). Intravenous artesunate (2·4 mg/kg of bodyweight) was administered as a rescue treatment if uncomplicated malaria progressed to severe malaria. The dosing of artesunate was scheduled according to WHO guidelines for severe malaria.^17^

In case of vomiting within the first 30 min after study drug administration, a full dose of the study drug was readministered; in case of vomiting between 30 min and 60 min post-administration, a half dose of the study drug was readministered. All patients were admitted to Kilifi County Hospital for at least 3 consecutive days and followed up at day 7 and, thereafter, weekly for up to 42 days. During each day of admission and each day of follow-up, a standardised symptom questionnaire, physical examination, and a measurement of vital signs were done. A 12-lead electrocardiograph was done at screening, baseline, hour 4, hour 24, hour 28, hour 48, and hour 52. For this study, the QTc interval was calculated by use of both Bazett's correction method and Fridericia's correction method (QTcF). Biochemistry and full blood count measurements were done at baseline, day 3, day 7, and day 28. If baseline biochemistry values were abnormal (grade 3 or 4) or the QTc interval was prolonged by more than 60 ms at any timepoint compared with baseline, arterolane–piperaquine–mefloquine or arterolane–piperaquine were discontinued and replaced by artemether–lumefantrine.

Asexual *P falciparum* parasite densities were assessed microscopically at screening, baseline, hours 4, 6, 8, and 12, and thereafter every 6 h until two consecutive blood films were negative. In every participant, parasite densities were also assessed at hours 24, 48, and 72 (even if two consecutive negative blood films were seen before these timepoints). A venous or capillary blood film was examined at each weekly follow-up until day 42 to detect recurrent infection. A recurrent infection was defined as blood smear positivity for asexual *P falciparum*. According to local guidelines, all recurrent infections were treated with artemether–lumefantrine. Whole blood was collected on dried blood spots at baseline and the day of recurrent infections.

Genetic markers of *P falciparum* resistance to artemisinin (*Pfkelch13* non-synonymous mutations) and piperaquine (*Pfplasmepsin2/3* gene amplification) were identified by use of the SPOTMalaria V2 platform, which uses a multiplexed amplicon sequencing method, implemented on Illumina sequencers. For PCR correction, DNA extraction and purification were done using the standardised kit (QIAamp DNA Mini Kit, Qiagen, Düsseldorf, Germany). Primer sequences, PCR amplification, and analyses were based on methods described previously.^21^ Recurrent infections were classified as a recrudescence if all *msp1, msp2*, and *glurp* alleles matched those that were present at baseline and as a reinfection if there were one or more allelic differences.^22^ Blood samples for pharmacokinetic measurements were obtained 0·5 h, 2·0 h, 6·0 h, 18·0 h, and 48·0 h after baseline in half the patients and 1·0 h, 3·0 h, 12·0 h, 24·0 h, and 72·0 h after baseline in the other half of patients. Plasma samples were shipped on dry ice to Sun Pharmaceutical Industries (the Clinical Pharmacology and Pharmacokinetics Unit) in Gurugram, India, for the assessment of plasma arterolane concentrations using a validated liquid chromatography with tandem mass spectrometry method.

### Outcomes

The primary endpoint was the 42-day PCR-corrected efficacy, defined as the absence of treatment failure in the first 42 days after treatment, of arterolane–piperaquine–mefloquine versus artemether–lumefantrine. The PCR-corrected efficacy denotes the absence of recrudescent infections during follow-up. The PCR-uncorrected efficacy denotes the absence of recrudescence and reinfections during follow-up. The 42-day PCR-corrected efficacy of arterolane–piperaquine–mefloquine versus arterolane–piperaquine was an important secondary endpoint.

Other prespecified secondary endpoints were parasite clearance half-lives, slide-positive parasitaemia at day 3, fever clearance times, 28-day PCR-corrected efficacy and 28-day and 42-day PCR-uncorrected efficacy, the proportion of patients completing a full treatment course, vomiting rates within 1 h of study drug administration, the prevalence of adverse events and serious adverse events, changes in heart rate at any timepoint, prolongation of the QTc interval (>60 ms or >500 ms) at hours 4, 24, 28, 48, and 52, and the pharmacokinetic profile of arterolane. Detailed results from the genomics and transcriptomics analyses will be reported separately.

Serious adverse events were reported to the sponsor, the appropriate ethics committees, the regulator, and an independent data and safety monitoring board within 24 h of awareness by the study team. Serious adverse events were defined as per the International Council for Harmonisation of Technical Requirements for Registration of Pharmaceuticals for Human Use guidelines for good clinical practice. The data and safety monitoring board met before the start of the trial and evaluated unblinded safety data after recruitment of 30 patients and then 100 patients. All adverse events were graded according to the Division of Acquired Immune Deficiency Syndrome Table for Grading the Severity of Adult and Paediatric Adverse Events (version 2.1; March, 2017), in which grade 1 is mild, grade 2 is moderate, grade 3 is severe, and grade 4 is potentially life-threatening.^23^

### Statistical analysis

We hypothesised that the 42-day PCR-corrected efficacy of arterolane–piperaquine–mefloquine would be non-inferior to artemether–lumefantrine. Based on previous experience elsewhere in Africa, we assumed that the efficacy of artemether–lumefantrine in Kenya was 98%.^14^, ^24^ WHO guidelines state that a change of first-line treatment should be considered if the efficacy of the first-line treatment is 90% or less. Therefore, we chose a −7% non-inferiority margin for arterolane–piperaquine–mefloquine versus artemether–lumefantrine. With this non-inferiority margin, a power of 80%, and a one-sided significance level of 0·025, a sample size of 63 patients per group was needed. Enrolling 73 patients per group allowed for a 15% loss to follow-up. In a secondary analysis, the efficacy of arterolane–piperaquine–mefloquine was compared with that of arterolane–piperaquine. Efficacy is reported as proportions. In addition, efficacy is reported as recrudescent and recurrent infection-free survival by use of Kaplan-Meier survival methods. We compared efficacy between the study groups using Fisher's exact test. Effect sizes are given as absolute differences or hazard ratios (HRs) with 95% CIs. Non-inferiority was assessed by constructing a two-sided 95% CI on the difference between arterolane–piperaquine–mefloquine and either of the non-triple combinations. Non-inferiority was concluded if the lower bound of the 95% CI did not exceed the non-inferiority margin of −7% of the risk difference. We analysed our primary outcome, safety, and secondary outcomes in the intention-to-treat population, which comprised all patients who received at least one dose of a study drug. Patients requiring rescue treatment with intravenous artesunate or who had a PCR-unclassified recurrent *P falciparum* infection were included in the treatment failure group in the intention-to-treat analysis. Patients who presented with a malaria reinfection, withdrew consent, or were lost to follow-up were included in the treatment success group in the intention-to-treat analysis. Patients with any of these events or in whom the study drug was replaced by artemether–lumefantrine (eg, because of a prolonged QTc interval) were excluded from the per-protocol analysis and were censored from the Kaplan-Meier survival analysis. We repeated our analyses of the primary outcome and secondary efficacy outcomes in the per-protocol population.

Parasite clearance half-lives were estimated by use of the Worldwide Antimalarial Resistance Network's parasite clearance estimator.^25^ The prevalence of adverse events related to symptoms, physical examination, and laboratory abnormalities were compared by use of descriptive statistics. Changes in heart rate and QTc intervals were compared by use of the unpaired *t* test. The incidences of vomiting within the first hour after drug administration were compared between study groups by use of a χ^2^ test. p-values are given and statistical significance was declared at 5%. All aforementioned analyses were done in Stata, version 15.

Collected pharmacokinetic data were analysed by use of a non-compartmental approach in Pkanalix, version 2019R2, to assess differences in the pharmacokinetic profile of arterolane with or without mefloquine coadministration. We assumed that arterolane, which is not locally available, was undetectable at baseline (at 0 h) and concentrations less than the lower limit of quantification were replaced with a value equal to half the lower limit of quantification. Because of the sparse sampling design, a non-compartmental analysis was done on the median concentration at each sampling timepoint (naive pooled analysis) comparing the children receiving arterolane–piperaquine with the children receiving arterolane–piperaquine–mefloquine. The analysis was done only after the first dose. The maximum concentration (C_max_) and the time to reach the maximum concentration (T_max_) were derived from the observed data. The terminal elimination rate constant (λ) was estimated by use of the software's best fit functionality (based on an adjusted R^2^ value and a uniform weighing). The elimination half-life was calculated as ln(2/λ). Exposure (area under the concentration time curve [AUC]) was calculated with the trapezoidal method by use of the linear method for ascending concentrations and the log-linear method for descending concentrations. Exposure was calculated to the last timepoint (AUC_last_) and, by use of λ to extrapolate from the last observed concentration, to infinity (AUC_∞_). Standard equations were used to calculate apparent elimination clearance and apparent volume of distribution. To evaluate the potential pharmacokinetic differences between arterolane, piperaquine, and mefloquine, concentrations at each sampling timepoint (including the 48 h and 72 h samples) were compared between groups by use of a Mann-Whitney *U*-test in GraphPad Prism, version 8.2.1. A data and safety monitoring board evaluated unblinded safety data after recruitment of 30 patients and then 100 patients. The study is registered in ClinicalTrials.gov, NCT03452475.

### Role of the funding source

Arterolane concentrations were measured and financed by Sun Pharmaceutical Industries (Gurugram, India), masked to the treatment group. Arterolane–piperaquine (Synriam) was provided for the study by Sun Pharmaceutical Industries. Other study drugs were purchased against their commercial value. The funders of the study had no role in study design, data collection, data analysis, data interpretation, or writing of the report.

## Results

**Figure 1:**
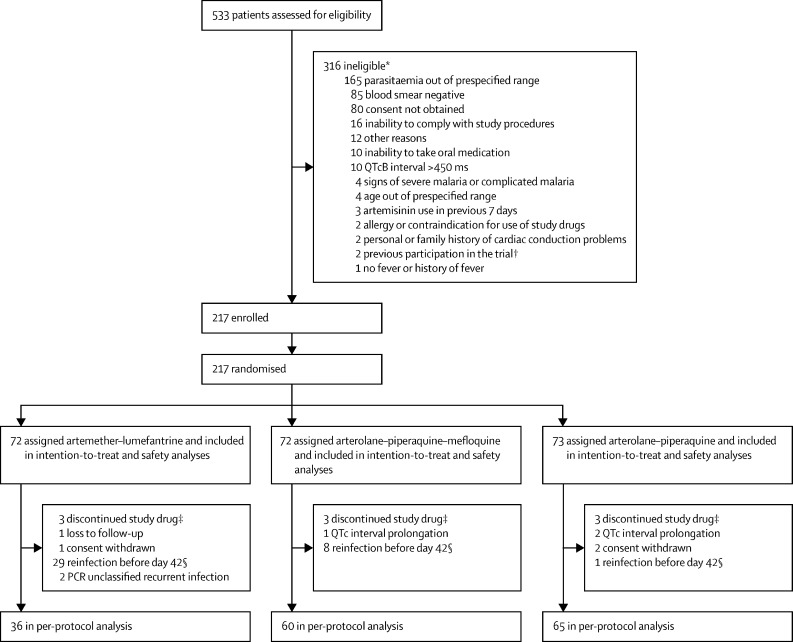
Trial profile QTcB interval=QT interval corrected for heart rate by use of Bazett's formula. *Reasons for exclusion are not exclusive. Some patients fulfilled more than one exclusion criterion. †After enrolment, randomisation, and administration of the first study drug dose (artemether–lumefantrine), it was found that one patient had been enrolled for a second time (one patient was directly excluded at screening as they had participated in the trial earlier). This patient was not included in the analysis. ‡The study drugs were discontinued in nine patients because of laboratory abnormalities at baseline, as per protocol. These nine patients were included in the intention-to-treat analysis and were excluded from the per-protocol analysis. §Patients with a reinfection at day 42 were included in the per-protocol analysis as a treatment success. A total of six reinfections (one in the artemether–lumefantrine group; two in the arterolane–piperaquine–mefloquine group; three in the arterolane–piperaquine group) occurred in patients that were excluded from the per-protocol analysis because their drugs were discontinued due to QTc interval prolongation (n=2) or baseline laboratory abnormalities (n=4).

Between March 7, 2018, and May 2, 2019, 533 children with an initial rapid diagnostic test positive for *P falciparum* were screened (figure 1). Of these, 217 patients were enrolled in the trial and randomly assigned to receive either arterolane–piperaquine (n=73), arterolane–piperaquine–mefloquine (n=72), or artemether–lumefantrine (n=72). All 217 patients were included in the intention-to-treat and safety analyses. 56 patients were excluded from the per-protocol analysis (figure 1). The median age of all 217 patients was 7·1 years (IQR 4·6–9·6) and just over half were male (table 1). Baseline characteristics were similar between the three study groups (table 1). Throughout the trial, 59 recurrent infections were identified, of which 56 were reinfections, two were unclassified (PCR correction was not possible because of a low DNA sample concentration at the day of recurrence), and one was a recrudescent infection. Of the 56 patients with reinfections, 38 were reinfected before day 42 and were excluded from the per-protocol analysis, 12 were reinfected on day 42, and six were reinfected after having stopped their study drug (figure 1).

**Table 1:** Baseline characteristics in the intention-to-treat population

		**Artemether–lumefantrine (n=72)**	**Arterolane–piperaquine–mefloquine (n=72)**	**Arterolane–piperaquine (n=73)**	**Total (n=217)**
Sex					
	Female	29 (40%)	34 (47%)	42 (58%)	105 (48%)
	Male	43 (60%)	38 (53%)	31 (42%)	112 (52%)
Median age, years		7·6 (4·9–9·1)	7·6 (4·3–9·8)	6·7 (4·1–9·5)	7·1 (4·6–9·6)
Mean tympanic temperature, °C		37·5 (1·2)	37·5 (1·2)	37·4 (1·1)	37·5 (1·2)
Weight, kg		18·8 (4·9)	19·4 (6·3)	18·6 (5·9)	18·9 (5·7)
Height, cm		114·7 (13·5)	114·4 (18·2)	112·9 (17·0)	114·0 (16·3)
Median heart rate, beats per min		118 (103–131)	118 (104–131)	119 (110–130)	119 (105–131)
Median respiratory rate, breaths per min		29 (26–32)	28 (26–34)	29 (25–32)	29 (26–32)
Median systolic blood pressure, mmHg		107 (100–117)	111 (103–117)	109 (101–118)	109 (101–117)
Median diastolic blood pressure, mmHg		66 (62–74)	70 (64–77)	69 (64–77)	69 (63–77)
QTcB interval, ms		420·1 (15·3)	418·9 (16·3)	419·2 (14·3)	419·4 (15·2)
QTcF interval, ms		376·8 (16·9)	376·5 (19·4)	374·0 (17·2)	375·8 (17·8)
Haematocrit, %		30·8% (6·0)	31·0% (3·7)	31·3% (4·2)	31·0% (4·7)
Geometric mean parasite count per μL*		61 683 (91 825; 848–358 726)	34 305 (79 305; 384–326 020)	52 508 (92 867; 80–571 530)	47 999 (89 216; 80–571 530)
Gametocytaemia		3 (4%)	1 (1%)	2 (3%)	6 (3%)
Bed net use in night before enrolment		43 (60%)	49 (68%)	51 (70%)	143 (66%)

Data are n (%), median (IQR), mean (SD), or mean (SD; range). QTcB interval=QT interval corrected for heart rate by use of Bazett's formula. QTcF interval=QT interval corrected for heart rate by use of Fridericia's formula.

* In some cases, the baseline parasitaemia concentration is outside the screening cutoff range because the parasitaemia decreased or increased between screening and baseline.

**Figure 2:**
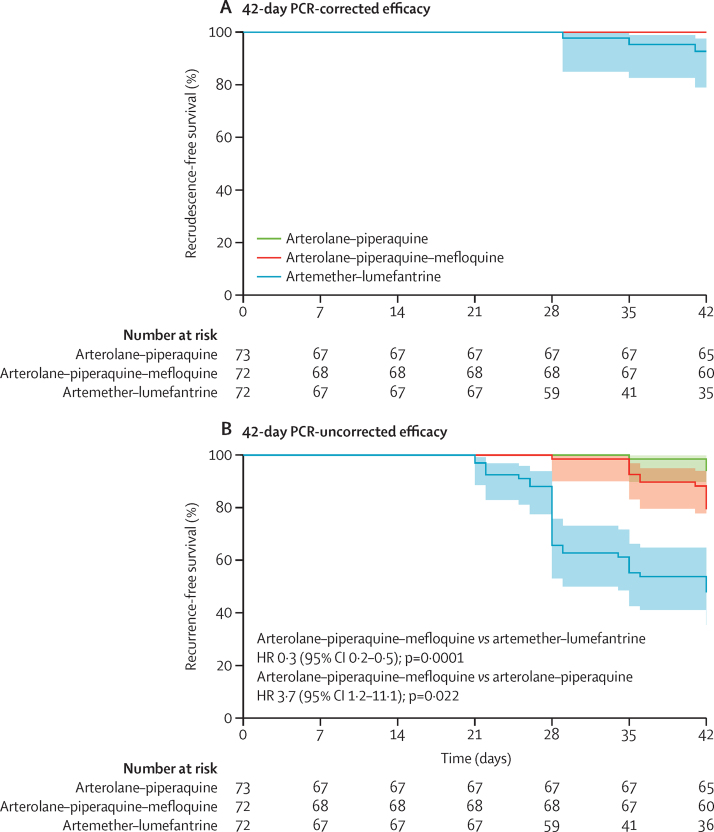
Kaplan-Meier survival curves by treatment group 42-day Kaplan-Meier survival estimates are shown for the time to *Plasmodium falciparum* recrudescent (A) and recurrent (B) infections following treatment with artemether–lumefantrine, arterolane–piperaquine–mefloquine, and arterolane–piperaquine. No meaningful HR was obtained for42-day PCR-corrected efficacy because one group had a number of participants with the event, but the other two groups had no or only one event. HR=hazard ratio.

In the intention-to-treat analysis, the 42-day PCR-corrected efficacy was 100% (95% CI 95–100; 73/73) for patients treated with arterolane–piperaquine, 100% (95–100; 72/72) for patients treated with arterolane–piperaquine–mefloquine, and 96% (88–99; 69/72) for patients treated with artemether–lumefantrine (table 2; appendix p 2). The 42-day PCR-corrected efficacy for arterolane–piperaquine–mefloquine was non-inferior to that of artemether–lumefantrine as the lower limit of the 95% CI of the risk difference did not cross the −7% non-inferiority margin (risk difference 4%, 95% CI 0–9; p=0·25). Kaplan-Meier survival analyses showed similar results (figure 2; appendix p 5). Furthermore, the 42-day PCR-corrected efficacy of arterolane–piperaquine–mefloquine was non-inferior to that of arterolane–piperaquine (table 2).

**Table 2:** 42-day and 28-day PCR-corrected and PCR-uncorrected efficacy according to antimalarial treatment in the intention-to-treat and per-protocol populations

	**Artemether–lumefantrine**	**Arterolane–piperaquine–mefloquine**	**Arterolane–piperaquine**	**Risk difference (95% CI); p value**	
				Arterolane–piperaquine–mefloquine versus artemether–lumefantrine	Arterolane–piperaquine–mefloquine versus arterolane–piperaquine
**Efficacy at day 42**					
PCR-corrected in the intention-to-treat population	69/72 (96%, 88 to 99)	72/72 (100%, 95 to 100)	73/73 (100%, 95 to 100)	4 (0 to 9); 0·25	0
PCR-uncorrected in the intention-to-treat population	36/72 (50%, 38 to 62)	56/72 (78%, 66 to 86)	66/73 (90%, 81 to 96)	28 (13 to 43); 0·0009	−12 (−24 to −1); 0·043
PCR-corrected in the per-protocol population	35/36 (97%, 86 to 100)	60/60 (100%, 94 to 100)	65/65 (100%, 95 to 100)	3 (−3 to 8); 0·38	0
PCR-uncorrected in the per-protocol population	32/65 (49%, 37 to 62)	54/68 (79%, 68 to 88)	62/66 (94%, 85 to 98)	30 (15 to 46); 0·0003	−15 (−26 to −3); 0·021
**Efficacy at day 28**					
PCR-corrected in the intention-to-treat population	72/72 (100%, 95 to 100)	72/72 (100%, 95 to 100)	73/73 (100%, 95 to 100)	0	0
PCR-uncorrected in the intention-to-treat population	48/72 (67%, 55 to 77)	69/72 (96%, 88 to 99)	70/73 (96%, 89 to 99)	29 (17 to 41); <0·0001	0 (−7 to 6); 1·0
PCR-corrected in the per-protocol population	44/44 (100%, 92 to 100)	67/67 (100%, 95 to 100)	67/67 (100%, 95 to 100)	0	0
PCR-uncorrected in the per-protocol population	42/65 (65%, 52 to 76)	67/68 (99%, 92 to 100)	66/66 (100%, 95 to 100)	34 (22 to 46); <0·0001	−2 (−4 to 1); 1·0

Data are n/N (%, 95% CI), unless otherwise specified. p values were calculated by use of two-sided Fisher's exact tests.

The two patients with recurrent infections for which PCR correction was not possible were treated with artemether–lumefantrine. These infections were interpreted conservatively as recrudescent, although it is more probable that these were actually reinfections with *P falciparum*. When reconsidering and imputing these two patients as having reinfections, the 42-day PCR-corrected efficacy of artemether–lumefantrine increases to 99% (95% CI 93–100; 71/72).

In the intention-to-treat population, the 42-day PCR-uncorrected efficacy was 90% for arterolane–piperaquine, 78% for arterolane–piperaquine–mefloquine, and 50% for artemether-lumefantrine (table 2; appendix p 2), reflecting a shorter post-treatment prophylactic effect conferred by lumefantrine. The 42-day PCR-uncorrected efficacy of arterolane–piperaquine–mefloquine was non-inferior to that of artemether–lumefantrine (table 2), whereas the 42-day PCR-uncorrected efficacy of arterolane–piperaquine–mefloquine was inferior to that of arterolane–piperaquine (table 2). However, in the Kaplan-Meier analysis, the CIs of the efficacy of these two groups overlap (figure 2). The 28-day PCR-corrected efficacy of arterolane–piperaquine–mefloquine was non-inferior to that of artemether–lumefantrine and that of arterolane–piperaquine (table 2; appendix p 3). The results from the per-protocol analysis confirmed the results from the intention-to-treat analysis (table 2).

**Figure 3:**
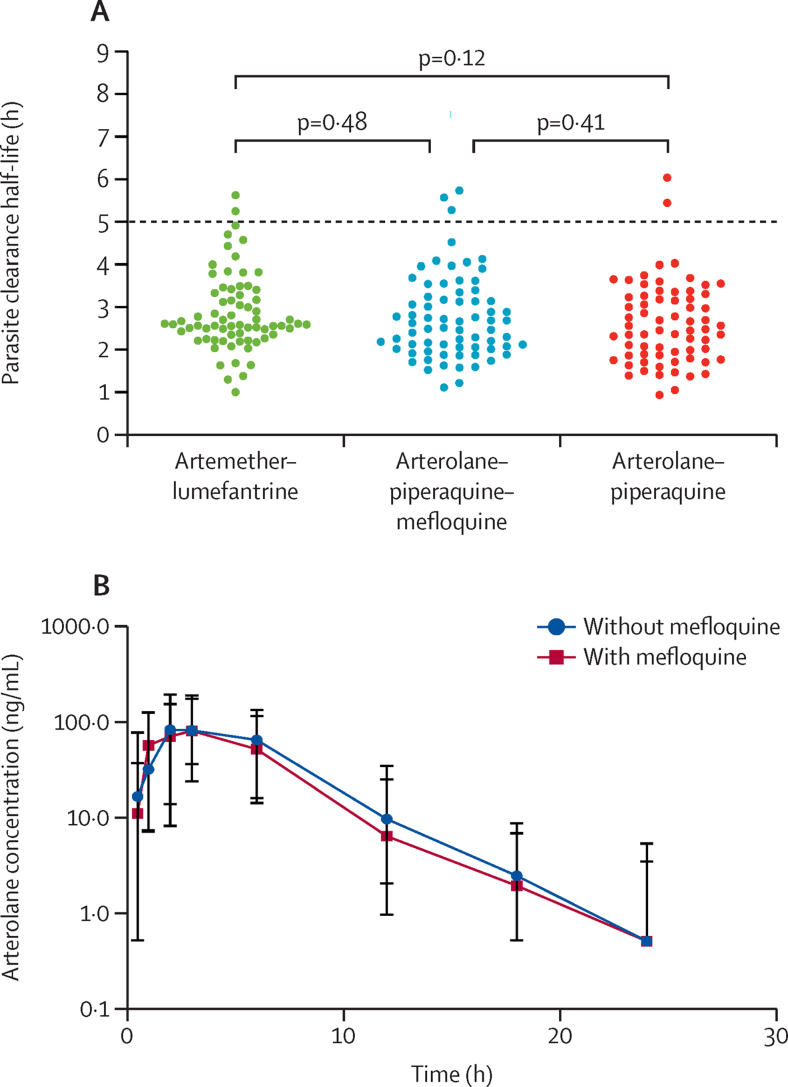
Parasite clearance half-lives and arterolane pharmacokinetics (A) Parasite clearance half-lives by study group. Each individual dot represents an individual patient's parasite clearance half-life after treatment with artemether–lumefantrine, arterolane–piperaquine–mefloquine, or arterolane–piperaquine. Reference bars indicate the mean value for each study group. The red dashed line indicates a half-life of 5 h, a common cutoff value for the delayed clearance phenotype. Comparisons were done by use of an unpaired t-test. (B) Pharmacokinetic concentration–time profiles of arterolane at an oral dose of 4 mg/kg, given in combination with piperaquine–mefloquine or piperaquine alone. The markers represent the median concentrations and the bars represent the 5–95th percentiles within each sample collection timepoint.

Parasite clearance half-lives could be calculated in 208 of 217 patients (figure 3A; appendix p 6). Half-lives were longer than 5 h in seven of 208 patients (3%, 95% CI 1–7). There was no significant difference between the mean parasite clearance half-lives in patients treated with arterolane–piperaquine (2·6 h, 95% CI 1·4–4·0), arterolane–piperaquine–mefloquine (2·7 h, 1·6–4·5), and artemether–lumefantrine (2·9, 1·6–4·7; figure 3A; appendix p 6). Slide-positive parasitaemia at day 3 was rare (n=2) and fever clearance times were similar between the three groups (appendix p 6).

We obtained *Pfkelch13* genotypes for 211 (97%) of 217 baseline samples. Of these, 203 (96%) did not carry any non-synonymous mutations. The Ala578Ser mutation, which is present throughout Africa and not associated with artemisinin resistance, was found in four samples. The other four samples were from mixed infections and contained rare, non-synonymous mutations (Asp399Asn, Ala486Ser, Cys542Arg, and Gly665Ser), whose effect on artemisinin sensitivity is unknown. Amplification of the *Pfplasmepsin*2/3 gene was found in none of the 103 samples in which amplification status could be determined.

The proportions of patients that completed a full treatment course were similar between treatments (appendix p 6). Reasons for discontinuation included baseline abnormalities in biochemistry results (n=9) and prolongation of the QTc interval (n=3).

Vomiting rates were significantly higher in patients treated with arterolane–piperaquine (p=0·0013) or arterolane–piperaquine–mefloquine (p=0·0006) than in patients treated with artemether–lumefantrine (table 3; appendix p 7). All patients were retreated successfully with an oral dose of the same drug combination. Vomiting rates were not significantly different between patients treated with arterolane–piperaquine and patients treated with arterolane–piperaquine–mefloquine (p=1·0; table 3; appendix p 7). The numbers of patients that vomited within the first hour after treatment at least once after enrolment in the trial were similar between arterolane–piperaquine treatment and arterolane–piperaquine–mefloquine treatment (p=1·0; table 3).

**Table 3:** Safety outcomes in the intention-to-treat population

	**Artemether–lumefantrine (n=72)**	**Arterolane–piperaquine–mefloquine (n=72)**	**Arterolane–piperaquine (n=73)**
Vomiting per number of treatments*	3/415 (1%, 0–2)	11/209 (5%, 3–9)	10/203 (5%, 2–9)
Vomiting at least once during the first h after treatment	3 (4%)	8 (11%)	9 (12%)
QTcB interval >60 ms more than baseline	0	1 (1%)	2 (3%)
QTcF interval >60 ms more than baseline	1 (1%)	13 (18%)	14 (19%)
QTcB interval >500 ms	0	0	0
QTcF interval >500 ms	0	0	0
Bradycardia (≤54 beats per min)	0	2 (3%)	1 (1%)

Data are n/N (%, 95% CI) or n (%). QTcB interval=QT interval corrected for heart rate by use of Bazett's formula. QTcF interval=QT interval corrected for heart rate by use of Fridericia's formula.

* Vomiting per number of treatments relates to observed vomiting within 1 h after drug administration. χ^2^ tests were used to compare vomiting rates (p=0·0006 for arterolane–piperaquine–mefloquine *vs* artemether–lumefantrine; p=0·0013 for arterolane–piperaquine *vs* artemether–lumefantrine; p=0·88 for arterolane–piperaquine–mefloquine *vs* arterolane–piperaquine).

Prolongation of the QTcB interval at hour 52, the time of expected peak concentrations of piperaquine, was greater after treatment with arterolane–piperaquine (mean increase 18·9 ms [SD 19·9]; p<0·0001) or arterolane–piperaquine–mefloquine (mean increase 15·7 ms [23·4]; p=0·0007) than after treatment with artemether–lumefantrine (mean increase 3·6 ms [16·3]; appendix pp 4, 8). Prolongation of the QTcF interval at hour 52 was greater after treatment with arterolane–piperaquine (mean increase 35·9 ms [SD 20·4]) or arterolane–piperaquine–mefloquine (mean increase 31·9 ms [25·8]) than after treatment with artemether–lumefantrine (mean increase 15·5 ms [16·9]; p<0·0001 for both). There was no significant difference in QTcB interval prolongation (p=0·29) or QTcF interval prolongation (p=0·68) at hour 52 between treatment with arterolane–piperaquine and treatment with arterolane–piperaquine–mefloquine (appendix p 4). QTc intervals for the other prespecified timepoints can be found in the appendix (p 8)**.** QTcF and QTcB intervals longer than 500 ms were not observed in any patient (table 3). The prevalence of a QTcB interval prolongation more than 60 ms than that at baseline was similar in patients treated with arterolane–piperaquine, arterolane–piperaquine–mefloquine, and artemether–lumefantrine (table 3). Changes in heart rate from baseline to hour 52 were similar between the arterolane–piperaquine group and the arterolane–piperaquine–mefloquine group (appendix pp 4, 8). Heart rate decreased more in the arterolane–piperaquine group (p=0·0007) and the arterolane–piperaquine–mefloquine group (p=0·024) than in the artemether–lumefantrine group (appendix pp 4, 8). The prevalence of bradycardia (heart rate ≤54 beats per min) was not different after treatment with arterolane–piperaquine, arterolane–piperaquine–mefloquine, or artemether–lumefantrine (table 3).

Headache, abdominal pain, and symptoms of upper respiratory tract infection were the most frequently reported adverse events (table 4). Overall, the prevalences of clinical adverse events (excluding upper respiratory tract complaints) were similar for patients treated with arterolane–piperaquine and patients treated with arterolane–piperaquine–mefloquine (table 4). Mild-to-moderate headache was reported by patients treated with artemether–lumefantrine at a higher frequency than by patients in the other treatment groups, which resulted in a higher total of adverse events for patients treated with artemether–lumefantrine than for patients treated with arterolane–piperaquine or arterolane–piperaquine–mefloquine (table 4).

**Table 4:** Adverse events and safety outcomes according to antimalarial treatment in the intention-to-treat population

	**Artemether–lumefantrine (n=72)**		**Arterolane–piperaquine–mefloquine (n=72)**		**Arterolane–piperaquine (n=73)**	
	Grades 1–2	Grades 3–4	Grades 1–2	Grades 3–4	Grades 1–2	Grades 3–4
**Symptoms**						
Upper respiratory tract complaints*	26 (36%)	0	19 (26%)	0	23 (32%)	0
Headache	13 (18%)	0	4 (6%)	0	5 (7%)	0
Fatigue	1 (1%)	0	0	0	0	0
Abdominal pain	7 (10%)	0	5 (7%)	0	5 (7%)	0
Loss of appetite	2 (3%)	0	1 (1%)	0	1 (1%)	0
Nausea	0	0	0	0	1 (1%)	0
Vomiting†	3 (4%)	0	1 (1%)	0	2 (3%)	0
Diarrhoea	2 (3%)	0	2 (3%)	0	2 (3%)	0
Itching	3 (4%)	0	4 (6%)	0	3 (4%)	0
Dizziness	1 (1%)	0	0	0	0	0
Blurred vision	0	0	0	0	0	0
Sleep disturbance	1 (1%)	0	0	0	0	0
Total‡	33 (46%)	0	17 (24%)	0	19 (26%)	0
**Laboratory abnormalities**						
Creatinine	47 (65%)	0	42 (58%)	1 (1%)	45 (62%)	0
Total bilirubin	3 (4%)	0	0	1 (1%)	1 (1%)	0
Alkaline phosphatase§	0/66	0/66	0/67	0/67	3/68 (4%)	0/68
Alanine aminotransferase	3 (4%)	0	3 (4%)	0	3 (4%)	1 (1%)
Aspartate aminotransferase	2 (3%)	0	5 (7%)	0	3 (4%)	0
γ-glutamyl transferase	6 (8%)	1 (1%)	5 (7%)	0	3 (4%)	0
Haemoglobin decrease (at hour 72, day 7, and day 28)¶	12 (17%)	1 (1%)	9 (13%)	1 (1%)	11 (15%)	1 (1%)
Leukopenia	1 (1%)	0	0	0	0	0
Neutropenia	6 (8%)	0	3 (4%)	0	1 (1%)	1 (1%)
Lymphopenia	0	0	0	0	0	0
Thrombopenia	3 (4%)	1 (1%)	4 (6%)	0	5 (7%)	2 (3%)

Data are n (%), n/N, or n/N (%).

* Upper respiratory tract complaints that were described as cough (if mild), conjunctivitis, nasopharyngitis, otitis media, rhinitis, rhinorrhoea, tonsillitis, and upper respiratory tract infections.

† The adverse event named vomiting relies on self-reporting, which could explain the observed discrepancy in the prevalence of vomiting and vomiting per number of treatments.

‡ Does not include upper respiratory tract complaints.

§ Alkaline phosphatase concentrations were not measured in the first 16 patients.

¶ The decrease is compared to the previous timepoint.

The most common biochemical adverse event was an abnormality in plasma creatinine concentrations (table 4). Mild-to-moderate increases in creatinine concentrations (not exceeding 0·94 mg/dL or 83 μmol/L) were found in 134 (62%) of 217 patients (table 4). This high proportion is probably related to the predefined, relatively low normal values for creatinine concentrations, which were not calibrated specifically for our study population. No difference in the prevalence of adverse events related to liver and renal toxicity was found between the study groups (table 4). None of the patients in this trial fulfilled Hy's criteria for liver toxicity (alanine aminotransferase or aspartate transferase concentrations >3 × the upper limit of normal and total bilirubin concentration >2 × the upper limit of normal). There were no differences between study groups in the prevalence of haematological adverse events and in the change in haemoglobin concentration (table 4).

A total of six serious adverse events fulfilling predefined criteria were reported, of which four were in patients treated with arterolane–piperaquine, one was in a patient treated with arterolane–piperaquine–mefloquine, and one was in a patient treated with artemether–lumefantrine (appendix p 9). Grade 4 thrombocytopenia occurred at day 3 (thrombocyte count 19 000 platelets per μL, which recovered to 241 000 platelets per μL by day 7) in a 4-year-old boy and was determined to be disease-related and unrelated to the study drug, arterolane–piperaquine. In three patients, two treated with arterolane–piperaquine and one treated with arterolane–piperaquine–mefloquine, QTcB interval prolongation compared with baseline exceeded 60 ms, resolved within 1 day, and was classified as definitely related to the study drugs. One patient treated with arterolane–piperaquine had a delayed discharge (day 4 instead of day 3; classified as possibly related) as parasite clearance was slow, although clinical recovery was rapid. One 4-year-old girl treated with artemether–lumefantrine was hospitalised for 2 days longer because of a urinary tract infection, which was classified as being unrelated to the study drug. A single dose of primaquine given at hour 24 was well tolerated.

The median concentration–time profiles for the two groups given arterolane are shown in figure 3B and the results from the non-compartmental analysis (ie, values for AUC∞, AUC_last_, C_max_, T_max_, the apparent elimination clearance, the apparent volume of distribution, and the elimination half-life) are shown in the appendix (p 9). The results indicated no substantial differences in overall arterolane exposure between patients receiving arterolane–piperaquine compared with those receiving arterolane–piperaquine–mefloquine (figure 3B). Comparing concentrations at each protocol timepoint, by treatment group, showed no significant difference in arterolane concentrations, except for lower arterolane concentrations at hour 72 after arterolane–piperaquine–mefloquine versus arterolane–piperaquine (p=0·0091; appendix p 10).

## Discussion

To our knowledge, this clinical trial is the first to compare the efficacy and safety of the triple antimalarial combination therapy, arterolane–piperaquine–mefloquine, with arterolane–piperaquine and artemether–lumefantrine for the treatment of uncomplicated falciparum malaria in Kenyan children. In addition, the study used a new weight-based dosing schedule for arterolane–piperaquine. The efficacy of arterolane–piperaquine–mefloquine was non-inferior to that of artemether–lumefantrine and arterolane–piperaquine, and all three treatment combinations were well tolerated.

Related to the much shorter plasma half-life of lumefantrine (half-life 3–4 days) compared with mefloquine (half-life 10–20 days) and piperaquine (terminal plasma half-life 20–30 days), the 42-day PCR-uncorrected efficacy, which includes reinfections with *P falciparum*, was low for treatment with artemether–lumefantrine, as result of a short post-treatment prophylactic effect, as has been described previously.^26^ Arterolane exposure was similar between patients treated with arterolane–piperaquine and patients treated with arterolane–piperaquine–mefloquine. This result is reassuring, because a drug–drug interaction study in healthy volunteers observed a 25% decrease in exposure of the artemisinin derivative dihydroartemisinin after the addition of mefloquine to dihydroartemisinin–piperaquine.^27^ In both the arterolane–piperaquine and arterolane–piperaquine–mefloquine groups, vomiting rates were low, although higher than those observed after treatment with artemether–lumefantrine. For the individual patient, the slightly worse tolerability of arterolane–piperaquine and arterolane–piperaquine–mefloquine might be outweighed by their longer post-prophylactic effect compared with artemether–lumefantrine, which could result in fewer malaria episodes, especially in areas of high malaria transmission.

Prolongation of the QTc interval at hour 52 was significantly greater with arterolane–piperaquine or arterolane–piperaquine–mefloquine than with artemether–lumefantrine. Importantly, the addition of mefloquine to arterolane–piperaquine did not further increase the QTc interval, which supports similar observations comparing QTc intervals after the addition of mefloquine to dihydroartemisinin–piperaquine.^13^ The prolongation of the QTc interval after other piperaquine-containing antimalarials has been shown not to be associated with an increased risk of sudden death.^28^ The total prevalence of clinical adverse events was similar for patients treated with arterolane–piperaquine and patients treated with arterolane–piperaquine–mefloquine, but was higher for patients treated with artemether–lumefantrine. The prevalence of laboratory abnormalities was similar between the treatment groups.

Combining arterolane with two partner drugs in a triple therapy could preserve the efficacy of each individual drug, as the chance of parasites developing resistance to all three drugs is the product of the chance of developing resistance to each individual drug, assuming there are no interactions between resistance mechanisms. The cost of a triple antimalarial therapy will be slightly higher than the cost of a standard ACT. However, these increases in costs should be considered against the costs associated with the emergence of multidrug-resistant malaria, which would probably increase the morbidity and mortality of malaria and could set back successes in malaria control and elimination.

Like the artemisinins, synthetic ozonides contain an endoperoxide bridge considered necessary for their parasiticidal potency, and in-vitro studies have shown reduced parasiticidal potency of arterolane (OZ277) in *Pfkelch13*-mutated (artemisinin-resistant) strains, suggesting cross-resistance.^29^, ^30^ However, the slightly longer plasma half-life of arterolane (around 3 h) compared with dihydroartemisinin (<1·5 h) might prolong and thus increase its parasiticidal activity in artemisinin-resistant *P falciparum* infections.^31^ Trials evaluating triple arterolane-based combinations in the setting of artemisinin and partner drug resistance are pending. Arterolane–piperaquine is not yet recommended, nor prequalified, by WHO for the treatment of malaria.

Our study had several limitations. The unblinded design could have affected the assessment of adverse events and the attribution of relatedness to the study drugs. However, objective measures, such as parasite clearance half-lives, treatment efficacy, electrocardiograph readings, and laboratory outcomes, are very unlikely to have been affected by the unblinded design of our study. Because our study relied on self-reporting of symptoms and all participants were young children, it is probable that the prevalence of complaints is an underestimation. The number of patients recruited to our study was small, and it is possible that less frequent side-effects were not identified. Furthermore, the study was done in a hospital setting, and each drug dose was administered and observed by a staff member, which might have resulted in a higher study drug adherence than that expected in a non-supervised setting. The most common reasons for study exclusion were having a parasitaemia out of our prespecified range or a negative blood smear. Future studies could evaluate the use of these drugs in clinical settings where diagnosis relies on rapid diagnostic tests because of the unavailability of microscopy. Further studies assessing treatment adherence in a non-clinical, non-supervised setting are needed. In addition, the relevance of the increased rates of vomiting we observed with triple ACTs compared with artemether–lumefantrine, with respect to treatment efficacy, should be assessed. The addition of primaquine is unlikely to have affected the comparison of efficacy and safety between the three study groups because primaquine does not affect asexual-stage *P falciparum.*

In conclusion, dosed according to weight, the efficacy of arterolane–piperaquine–mefloquine was non-inferior to that of artemether–lumefantrine and arterolane–piperaquine, and all combinations were safe and well tolerated, in the treatment of uncomplicated falciparum malaria in Kenyan children.

## Data sharing

With publication, deidentified, individual participant data that underlie this Article, along with a data dictionary describing variables in the dataset, will be made available to researchers whose proposed purpose of use is approved by the Kenya Medical Research Institute Wellcome Trust Research Programme (KWTRP)data access committee. Data will be shared directly or through access on the Harvard Dataverse repository. To request the dataset directly, please send a signed data request form to dgc@kemri-wellcome.org. The data request form can be found online on the KWTRP Harvard Dataverse repository. Individual patient data will be available under managed access through the data access committee. There will be no restrictions on analysis; all requests will be evaluated by the data access committee for a decision in line with consent for data sharing and findability, accessibility, interoperability, and reusability (known as FAIR) principles. The mechanism will be open access and managed via email (dgc@kemri-wellcome.org) for the data requests to be considered by the data access committee. Related documents, such as the study protocol, statistical analysis plan, and informed consent form, will be made available (with publication) on request to the corresponding author (mhamaluba@kemri-wellcome.org). The data will be open access for the informed consent form, protocol, and statistical analysis plan. Once the data are in the Harvard Dataverse repository, a request for these documents can be made from the dataset page.

## Declaration of interests

We declare no competing interests.
