# Supplementary material for: Arterolane–piperaquine–mefloquine versus arterolane–piperaquine and artemether–lumefantrine in the treatment of uncomplicated Plasmodium falciparum malaria in Kenyan children: a single-centre, open-label, randomised, non-inferiority trial
Source: Lancet Infect Dis. 2021 Oct;21(10):1395–406. doi: 10.1016/S1473-3099(20)30929-4 (PMC8461080; doi:10.1016/S1473-3099(20)30929-4)
Supplement: Supplementary appendix [file mmc1.pdf]

THE LANCET
Infectious Diseases

Supplementary appendix

This appendix formed part of the original submission and has been peer reviewed. We post it as supplied by the authors.

Supplement to: Hamaluba M, van der Pluijm RW, Weya J, et al. Arterolane–piperaquine–mefloquine versus arterolane–piperaquine and artemether–lumefantrine in the treatment of uncomplicated *Plasmodium falciparum* malaria in Kenyan children: a single-centre, open-label, randomised, non-inferiority trial. *Lancet Infect Dis* 2021; published online June 7. https://doi.org/10.1016/S1473-3099(20)30929-4.

Supplementary figure 1. Location of Pingilikani dispensary and Kilifi County Hospital in Kenya

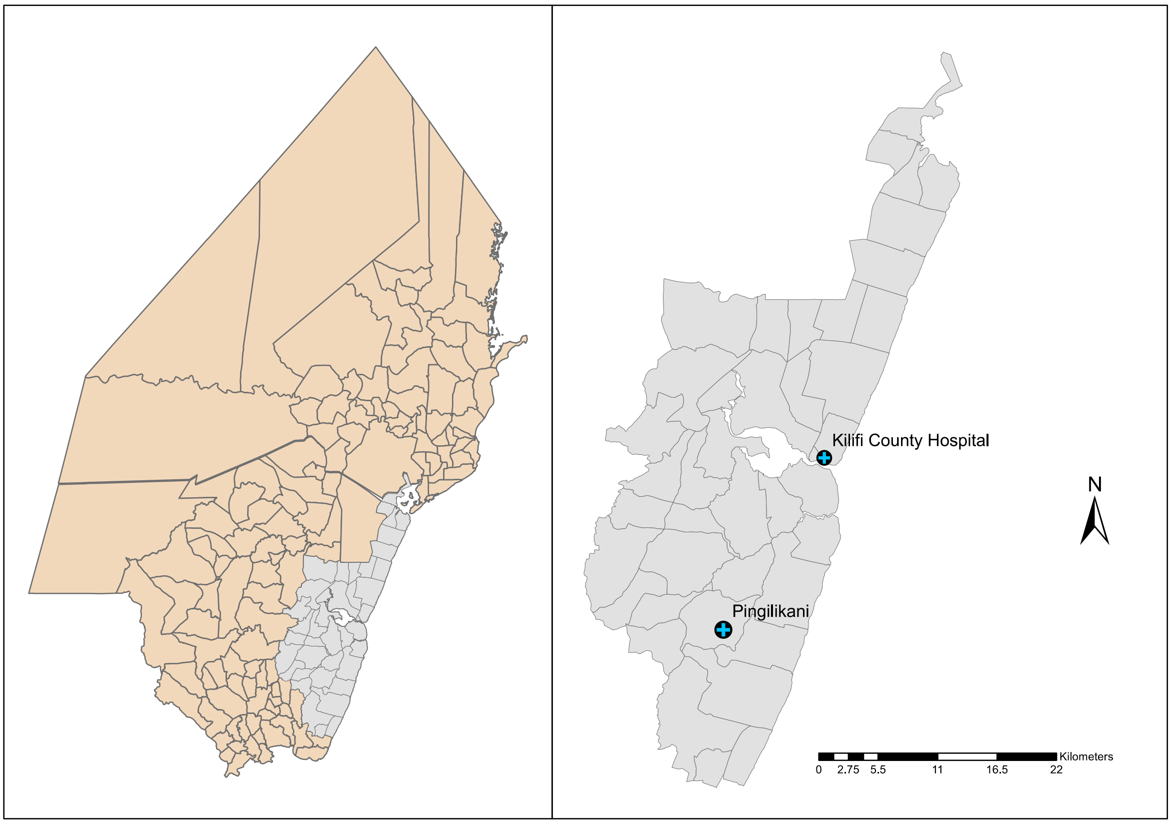

Supplementary figure 2. Comparison of day 42 PCR corrected and uncorrected efficacy by study arm

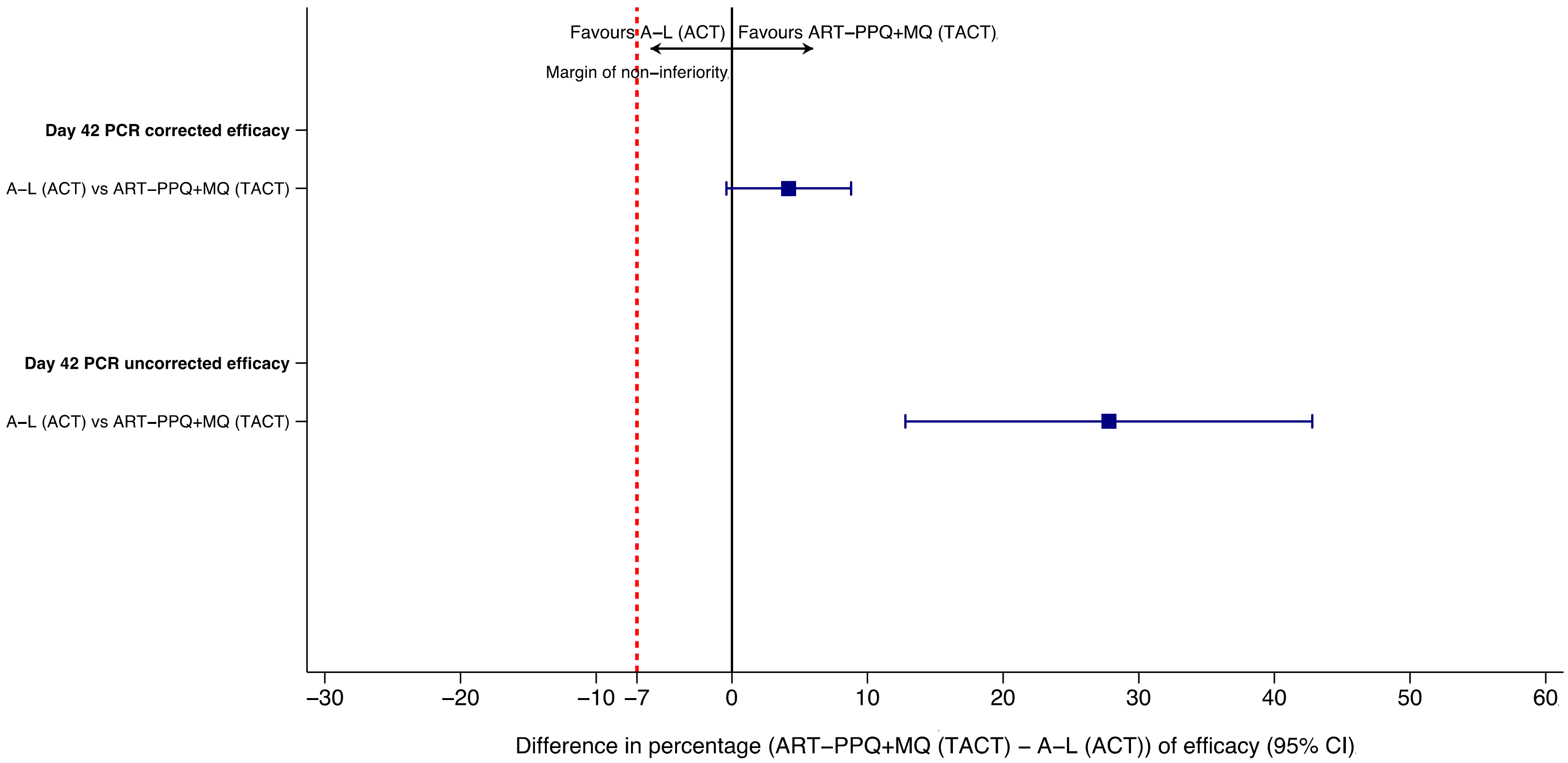

Comparison of 42 day efficacy (risk difference with 95% CI) after receiving artemether-lumefantrine (AL) or arterolane-piperaquine+mefloquine (ART-PPQ+MQ). The 42 day PCR corrected efficacy denotes the absence of recrudescent infections during the 42 days of follow-up. The 42 day PCR uncorrected efficacy denotes the absence of both recrudescence and reinfections with *P. falciparum* during the follow-up period.

Supplementary figure 3. Comparison of day 28 PCR corrected and uncorrected efficacy by study arm

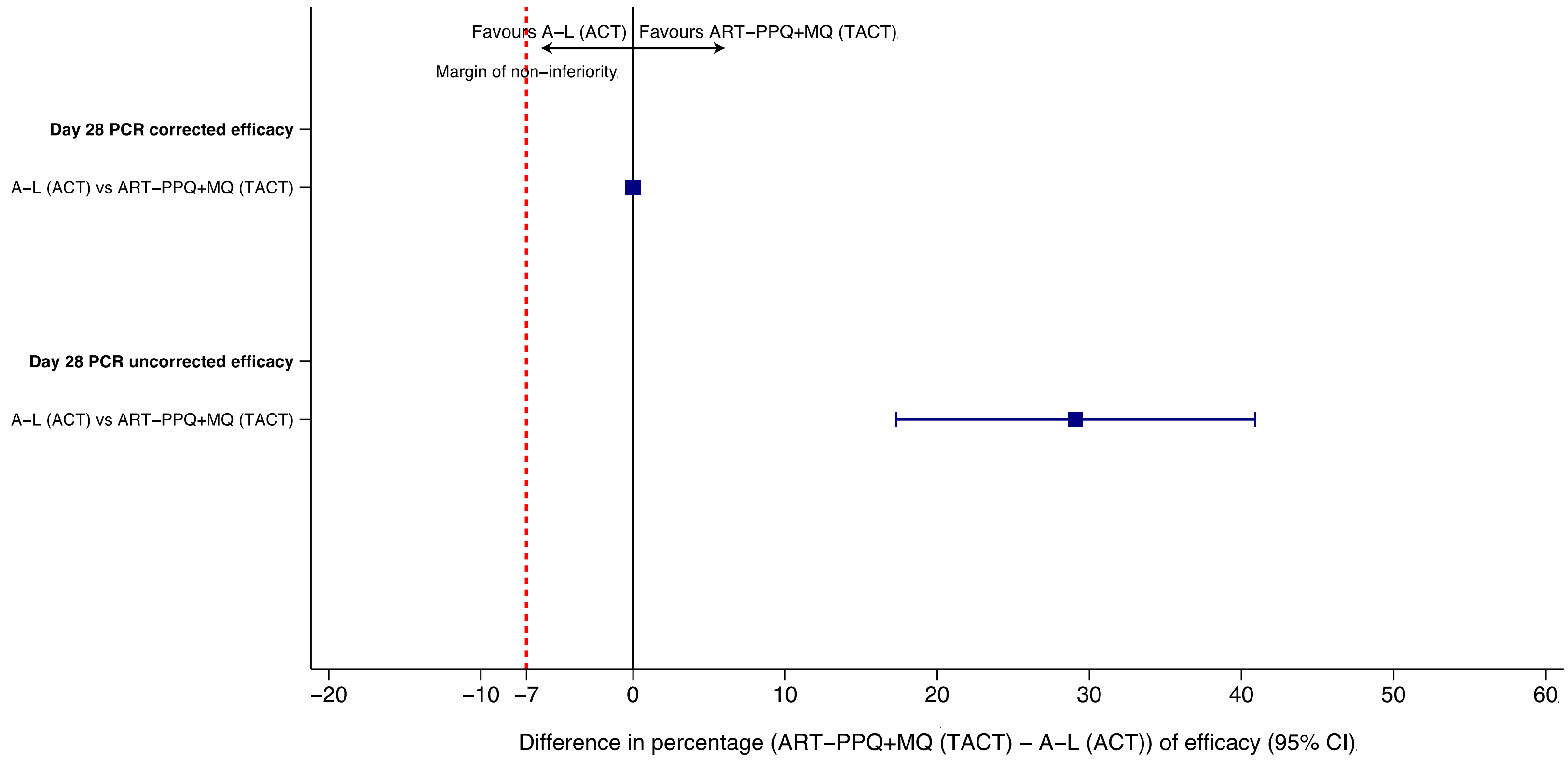

Comparison of 28 day efficacy (risk difference with 95% CI) after receiving artemether-lumefantrine (AL) or arterolane-piperaquine+mefloquine (ART-PPQ+MQ). The 28 day PCR corrected efficacy denotes the absence of recrudescent infections during the first 28 days of follow-up. The 28 day PCR uncorrected efficacy denotes the absence of both recrudescence and reinfections with *P. falciparum* during the first 28 days of follow-up.

Supplementary figure 4. Changes in QTcBazett-interval, QTc-Fridericia-interval and heart rates

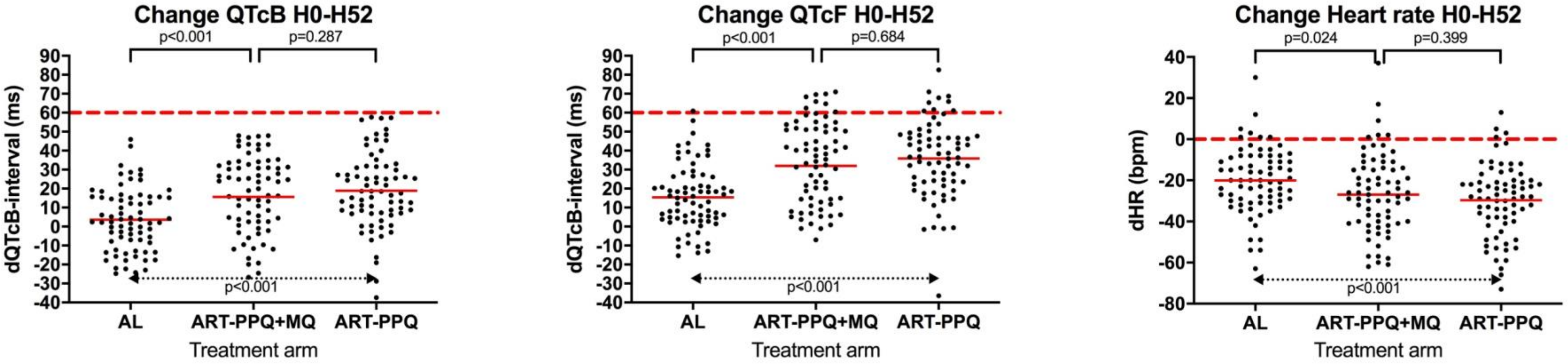

Each individual dot represents the change within each subject of the QTcBazett-interval (QTcB), QTcFridericia-interval (QTcF) and heart rate between baseline and hour 52 after arterolane-piperaquine (ART-PPQ), arterolane-piperaquine+mefloquine (ART-PPQ+MQ) and artemether-lumefantrine (AL). Hour 52 after baseline represents the timing of the expected peak values of piperaquine. Reference bars indicate mean changes. The red dashed line indicates a change in QTc-interval of 60 miliseconds, which was used as a safety cut-off in this trial. Comparisons were performed using an unpaired t-test.

Supplementary table 1. 42 day and 28 day efficacy (PCR corrected and uncorrected) using Kaplan Meier survival analysis

	Artemether-lumefantrine	Arterolane-piperaquine +mefloquine	Arterolane-piperaquine	Arterolane-piperaquine+mefloquine versus artemether-lumefantrine	Arterolane-piperaquine+mefloquine versus Arterolane-piperaquine
	% (95% CI)	% (95% CI)	% (95% CI)	Hazard ratio (95%CI) (p-value)	
PCR corrected efficacy at day 42	93<br>(79 to 98)	100<br>(NA)	100<br>(NA)	NA*	1 (NA) NA
PCR uncorrected efficacy at day 42	48<br>(36 to 59)	79<br>(68 to 87)	94<br>(85 to 98)	0.3(0.2 to 0.5)<br>(0·0001)	3.7(1.2 to 11.1) (0·022)
PCR corrected efficacy at day 28	100·0<br>(NA)	100·0<br>(NA)	100<br>(NA)	1 (NA)<br>NA	1 (NA)<br>NA
PCR uncorrected efficacy at day 28	66<br>(53 to 76)	99<br>(90 to 100)	100<br>(NA)	0.04(0.005 to 0.3)<br>(0·0015)	NA*

NA* no meaningful Hazard ratio is obtained because one arm has a number of participants with an event, but the other two arms have, no/or only one event. This phenomenon creates complexities for the Maximum Likelihood Estimation (MLE) algorithms and the estimated hazard ratios become infinite (too large).

Supplementary table 2. Parasite clearance characteristics, parasite genotypes and fever clearance time by arm

	Artemether-lumefantrine	Arterolane-piperaquine+mefloquine	Arterolane-piperaquine
Mean parasite clearance half-life in hours (CI 95%)*	2·9 (1·6-4·7)	2·7 (1·6-4·5)	2·6 (1·4-4·0)
Median parasite clearance half-life in hours (25th-75th percentile)	2·6 (2·4-3·3)	2·6 (2·1-3·2)	2·5 (1·9-3·3)
Parasite clearance half-life >5 hours (n/N, %, CI 95%)	2/70 (2·9) (0·3-9·9)	3/70 (4·3) (0·9-12·0)	2/68 (2·9) (0·4-10·2)
Median time to 50% parasite clearance in hours (25th-75th percentile)	5·9 (4·1-7·3)	6·3 (4·4-7·6)	5·5 (4·0-7·0)
Median time to 90% parasite clearance in hours (25th-75th percentile)	12·6 (9·8-14·5)	12·0 (10·0-13·5)	10·8 (9·2-13·2)
Day 3 positivity) (n/N, %, CI 95%)	1/73 (1·4) (0-7·4)	0/70 (0) (0-5·1)	1/73 (1·4) (0-7·4)
Pfkelch13 genotype: wild-type	66/67 (98·5) (92·0-99·9)	71/71 (0) (94·9-100)	66/73 (90·4) (81·2-96·1)
Pfkelch13 genotype: A578S or mixed infection wild-type/A578S	1/67 (0) (0-5·4)	0/71 (0) (0-5·1)	3/73 (4·1) (0·9-11·5)
Pfkelch13 genotype: Other non-synonymous mutations	0/67 (0) (0-5·4)	0/71 (0) (0-5·1)	4/73 (5·5) (1-5·13·4)
Pfplasmepsin2/3 gene amplification	0/31 (0) (0-11·2)	0/31 (0) (0-11·2)	0/41 (0) (0-8·6)
Median fever clearance time in hours (25th-75th percentile)	36 (24-48)	36 (24-48)	36 (24-48)
*Parasite clearance half-lives were not obtained in 9 patients.			

Supplementary table 3· Proportions of patients that completed a full course of treatment

	Artemether-lumefantrine	Arterolane-piperaquine +mefloquine	Arterolane-piperaquine	Arterolane-piperaquine +mefloquine vs Artemether-lumefantrine	Arterolane-piperaquine vs Artemether-lumefantrine	arterolane-piperaquine +mefloquine vs Arterolane-piperaquine
	n/N (%) (95% CI)	n/N (%) (95% CI)	n/N (%) (95% CI)	Difference (95%CI) (p-value)		
Completing full course of study drug	66/72 (91·7) (82·7 to 96·9)	68/72 (94·4) (86·4 to 98·5)	68/73 (93·2) (84·7 to 97·7)	2·7 (-5·6 to 11·0) 0·745	1·5 (-7·1 to 10·0) 0·765	1·2 (-6·6 to 9·0) 1·00

Supplementary table 4. Vomiting rates per timepoint within 1 hour study drug administration

	Artemether-lumefantrine	Arterolane-piperaquine+ mefloquine	Arterolane-piperaquine
	n=72	n=72	n=73
First hour after H0	1/72 (1·4)	7/72 (9·7)	5/73 (6·8)
First hour after H8	2/69 (2·9)	NA	NA
First hour after H24	0/69 (0)	4/69 (5·8)	3/70 (4·3)
First hour after H36	0/69 (0)	NA	NA
First hour after H48	0/69 (0)	0/68 (0)	2/66 (3·0)
First hour after H60	0/67 (0)	NA	NA
Total	3/415 (0·7)	11/209 (5·3)	10/203 (4·9)
Results are presented as vomiting number of administrations (n/N, %)			

Comparison of incidence of vomiting within 1 hour after drug administration
Artemether-lumefantrine versus arterolane-piperaquine+mefloquine=0·0006
Artemether-lumefantrine versus arterolane-piperaquine=0·0013
Arterolane-piperaquine versus arterolane-piperaquine+mefloquine=1·000

Supplementary table 5. QTc-intervals and changes in QTc-intervals over time

Time-point	Artemether-lumefantrine				Arterolane-piperaquine+mefloquine				Arterolane-piperaquine			
	QTcB	Δ-QTcB	QTcF	Δ-QTcF	QTcB	Δ-QTcB	QTcF	Δ-QTcF	QTcB	Δ-QTcB	QTcF	Δ-QTcF
H0	420·1 (15·3)	NA	376·8 (16·9)	NA	418·9 (16·3)	NA	376·5 (19·4)	NA	419·2 (14·3)	NA	374·0 (17·2)	NA
H4	421·1 (17·9)	1·0 (15·4)	381·5 (18·4)	4·8 (15·6)	431·2 (20·3)	12·2 (17·6)	394·6 (23·0)	18·0 (19·5)	435·8 (19·6)	16·6 (15·8)	396·3 (22·3)	22·3 (16·5)
H24	419·8 (14·7)	-0·2 (15·4)	384·1 (16·1)	7·4 (17·9)	426·1 (16·7)	7·2 (15·9)	395·0 (16·7)	18·5 (17·2)	425·4 (15·1)	6·2 (13·9)	391·2 (16·1)	17·2 (14·1)
H28	421·7 (15·7)	1·6 (16·7)	389·1 (16·7)	12·3 (17·5)	438·7 (28·8)	19·8 (28·5)	409·1 (25·7)	32·6 (24·7)	442·9 (19·6)	23·7 (19·4)	411·8 (18·9)	37·8 (18·9)
H48	420·6 (15·3)	0·5 (16·8)	388·8 (15·8)	12·1 (17·0)	424·0 (16·9)	5·1 (17·5)	396·4 (17·9)	19·9 (17·7)	424·2 (16·2)	5·0 (16·6)	394·5 (15·1)	20·5 (15·5)
H52	423·7 (14·6)	3·6 (16·3)	392·2 (16·6)	15·5 (16·9)	434·6 (22·6)	15·7 (23·4)	408·4 (26·4)	31·9 (25·8)	438·2 (18·1)	18·9 (19·9)	410·1 (20·1)	35·9 (20·4)
Data are provided as mean and standard deviation												

Supplementary table 6: Heart rates and changes in heart rates over time

Time-point	Artemether-lumefantrine		Arterolane-piperaquine+mefloquine		Arterolane-piperaquine	
	Heart rate	Δ-Heart rate	Heart rate	Δ-Heart rate	Heart rate	Δ-Heart rate
H0	117 (20)	NA	116 (21)	NA	121 (20)	NA
H4	110 (19)	-7 (14)	104 (18)	-12 (15)	108 (16)	-13 (14)
H24	104 (19)	-13 (15)	96 (18)	-20 (18)	101 (16)	-20 (14)
H28	99 (16)	-19 (15)	93 (17)	-24 (21)	94 (16)	-27 (16)
H48	98 (18)	-19 (17)	92 (20)	-24 (20)	94 (16)	-27 (17)
H52	97 (19)	-20 (16)	89 (19)	-27 (20)	91 (18)	-30 (17)
Data are provided as mean and standard deviation						

Supplementary table 7. Listing of Serious Adverse Events

SAE number	SAE description	Study arm	Age	Sex	Relatedness SAE to study drugs	Severity of SAE
1	Thrombocytopenia	Arterolane-piperaquine	4	Male	Not Related	Life-threatening
2	Prolonged QTc interval	Arterolane-piperaquine	4	Male	Definitely related	Severe
3	Prolonged QTc interval	Arterolane-piperaquine	6·7	Male	Definitely related	Severe
4	Prolonged parasite clearance	Arterolane-piperaquine	5·1	Female	Possibly related	Mild
5	Presumed urinary tract infection	Artemether-lumefantrine	4·2	Female	Not related	Moderate
6	Prolonged QTc interval	Arterolane-piperaquine+mefloquine	2·1	Female	Definitely related	Moderate

Supplementary table 8. Pharmacokinetic parameters of arterolane, using a naïve-pooled non-compartmental analysis

Parameter	Arterolane-piperaquine	Arterolane-piperaquine+mefloquine
AUC_{∞} (h×ng/mL)	592	518
AUC_{last} (h×ng/mL)	590	516
C_{max} (ng/mL)	83·3	80·9
T_{max} (h)	2·00	3·00
CL/F (L/h)	122	140
V/F (L)	499	663
$t_{1/2}$ (h)	2·83	3·29

AUC is the area under the concentration time curve, C_{max} is the maximum concentration, T_{max} is the time to reach the maximum concentration, CL/F is the apparent elimination clearance, V/F is the apparent volume of distribution, $t_{1/2}$ is the terminal elimination half-life.

Supplementary table 9. Comparison of arterolane drug concentrations at individual timepoints.

Protocol time (h)	Arterolane drug concentrations (ng/mL)[a]		
	Arterolane given alone, n	Arterolane given together with mefloquine, n	p-value[b]
0.5	16.7 (7.84-35.5), n=36	11.1 (3.17-22.4), n=36	0.072
1	32.1 (20.2-75.2), n=37	57.2 (24.8-75.2), n=36	0.27
2	83.3 (55.2-149), n=36	70.7 (44.1-97.0), n=36	0.11
3	81.8 (53.6-113), n=37	80.9 (63.2-116), n=36	0.73
6	65.0 (34.9-91.8), n=36	52.0 (34.8-76.5), n=36	0.51
12	9.72 (5.11-17.3), n=37	6.44 (2.57-14.1), n=36	0.084
18	2.48 (0.954-3.95), n=36	1.96 (0.946-4.43), n=36	0.85
24	0.515 (0.515-1.35), n=37	0.515 (0.515-0.515), n=35	0.14
48	2.07 (0.696-3.34), n=34[c]	1.33 (0.515-2.34), n=35[c]	0.083
72	2.23 (1.49-5.82), n=35[d]	1.19 (0.515-2.47), n=33[d]	0.0091

Data below the lower limit of quantification (1.03 ng/mL) were replaced with half the limit of quantification concentration (0.515 ng/mL). n is the number of subjects included in the analysis.

[a] Presented as median (25th-75th percentiles)

[b]p-value calculated with Mann-Whitney test

[c] Patients who received only one dose were excluded from the analysis at this timepoint.

[d] Patients who received only one or two doses were excluded from the analysis at this timepoint.

Study drug dosing schedules

**Arterolane maleate-piperaquine phosphate dosing schedule (administered at H0, H24 and H48)**		
Body weight (kg)	Arterolane-piperaquine (37·5/187·5 mg)	Arterolane-piperaquine (150/750 mg)
5-7·9	0·75	0
8-10·9	1·25	0
11-16·9	1·75	0
17-24·9	2·5	0
25-35·9	3·5	0
36-59·9	1	1
60-79·9	3	1
80-100	1	2

**Mefloquine dosing schedule (administered at H0, H24 and H48)**	
Body weight (kg)	Milliliter (50 mg/ml)
5-5·9	0·8 milliliter
6-6·9	1 milliliter
7-7·9	1·2 milliliter
8-8·9	1·3 milliliter
9-9·9	1·5 milliliter
10-10·9	1·7 milliliter
11-11·9	1·8 milliliter
	Tablets (250mg/tablet)
12-16·9	0·5 tablet
17-23·9	0·75 tablet
24-33·9	1 tablet
34-43·9	1·25 tablets
44-48·9	1·5 tablets
49-53·9	1·75 tablets
54-63·9	2 tablets
64-71·9	2·25 tablets
72-77·9	2·5 tablets
78-100	2·75 tablets

**Artemether-lumefantrine dosing schedule (administered at H0, H8, H24, H36, H48 and H60)**	
Body weight (kg)	Artemether-lumefantrine tablet 20mg/120mg
5-14·9	1 tablet
15-24·9	2 tablets
25-34·9	3 tablets
≥35	4 tablets

**Primaquine dosing schedule (administered at H24)**	
Age (months)	Primaquine dose base in mg
6-<12	1·25 mg
12-<72	2·5 mg
72-<120	5 mg
120-<180	7·5 mg
